# The effectiveness of hyperbaric oxygen therapy for managing radiation-induced proctitis – results of a 10-year retrospective cohort study

**DOI:** 10.3389/fonc.2023.1235237

**Published:** 2023-08-11

**Authors:** António Moreira Monteiro, Diogo Alpuim Costa, Virgínia Mareco, Carla Espiney Amaro

**Affiliations:** ^1^ Faculdade de Medicina, Universidade de Lisboa, Lisbon, Portugal; ^2^ NOVA Medical School, Faculdade de Ciências Médicas, Lisbon, Portugal; ^3^ Centro de Medicina Subaquática e Hiperbárica (CMSH), Armed Forces Hospital, Lisbon, Portugal; ^4^ Medical Oncology Department, Hospital de Cascais Dr. José de Almeida, Alcabideche, Portugal; ^5^ Hematology and Oncology Department, CUF Oncologia, Lisbon, Portugal; ^6^ Radiotherapy Department, Hospital de Santa Maria, Centro Hospitalar Universitário Lisboa Norte, Lisbon, Portugal

**Keywords:** radiotherapy, radiation injury, late radiation tissue injury, radiation-induced proctitis, radiation proctitis, hyperbaric oxygenation, hyperbaric oxygen, hyperbaric oxygen therapy

## Abstract

**Introduction:**

Despite modern radiotherapy (RT) techniques, radiation-induced proctitis (RIP) remains a significant complication of RT for pelvic organ malignancies. Over the last decades, an enormous therapeutic armamentarium has been considered in RIP, including hyperbaric oxygen therapy (HBOT). However, the evidence regarding the impact of HBOT on RIP is conflicting. This study aims to evaluate the effectiveness and safety of HBOT in the treatment of RIP.

**Methods:**

Ten-year (2013-2023) retrospective analysis of all consecutive patients with RIP treated with HBOT at Centro de Medicina Subaquática e Hiperbárica (CMSH) (Armed Forces Hospital – Lisbon, Portugal). Patients were exposed to 100% oxygen at 2.5 ATA, in a multiplace first-class hyperbaric chamber, for 70-min periods, once daily, five times per week. Fisher’s exact test was performed using SPSS (version 23.0); p<0.05 was accepted as statistically significant.

**Results:**

Of a total of 151 patients with RIP, 88 were included in the final analysis, of whom 38.6% evidenced other concurrent radiation-induced soft tissue lesions. The most reported primary pelvic tumor treated with RT was prostate cancer (77.3%), followed by cervical cancer (10.2%). Hematochezia was the most observed clinical manifestation (86.4%). After a median of 60 HBOT sessions (interquartile range [IQR]: 40-87.5), 62.5% and 31.8% of patients achieved a clinical complete and partial response, respectively, with a hematochezia resolution rate of 93.7% (complete or partial). While partial and complete responses require fewer than 70 sessions of HBOT in terms of overall RIP symptoms (p=0.069), isolated hematochezia tends to require at least 70 sessions (p=0.075). Individuals with at least two concurrent late radiation tissue injuries were associated with a complete response to HBOT (p=0.029). Only about 5.7% of patients did not respond to the treatment. Eighteen patients (20.5%) developed reversible ear barotrauma. The number of HBOT sessions was a predictor of HBOT side effects (odds ratio: 1.010; 95% confidence interval, 1.000-1.020; p=0.047).

**Conclusion:**

The HBOT proved to be an effective and safe treatment for RIP refractory to medical and/or endoscopic treatments. This real-world evidence study adds value to published data on the management of RIP with HBOT.

## 1 Introduction

Cancer is a foremost cause of global morbidity and mortality (1–4). The International Agency for Research on Cancer predicts a 47% rise in the global cancer burden in 2040 compared to 2020, expecting 28.4 million cancer cases (5). Pelvic organ cancer designates any malignant tumor primarily originating in any pelvic cavity organ (6, 7). Despite the therapeutic avenue innovation, radiotherapy (RT) remains one of the cornerstones for managing malignant tumors, with 50% of patients receiving RT during their course of illness (8–10). Introducing new RT techniques (e.g., three-dimensional conformal radiation therapy and intensity-modulated radiation therapy), it has been possible to eradicate malignant tumor cells with minimal exposure to surrounding tissues (11–13). However, these modern RT approaches are not exempted from side effects, late radiation-induced proctitis (RIP) being one of them (9, 14).

The RIP is defined as an iatrogenic lesion of the mucosa and submucosa of the rectum or rectosigmoid transition following RT for pelvic cancer (8, 10). It can be classified into acute or chronic RIP, considering the time from the end of RT protocol to the onset of RT-related clinical signs and symptoms (8, 15). The incidence rate is estimated from 2 to 39% (16). Proctitis starts with an initial mucosal injury induced by ionizing radiation, and the organism reacts through a chronic inflammatory response leading to fibrosis, obliterative endarteritis, tissue hypoxia and ischemia, and neoangiogenesis (17, 18). Gut microbiota and oxidative stress may also be important in chronic radiation enteritis and colitis (19–21). Different clinical manifestations may develop, including proctalgia, diarrhea, constipation, recurrent ileus, mucus discharge, hematochezia, ulceration/fistulae formation, fecal incontinence/urgency, or a predisposition to a malabsorption syndrome. Patients may also experience other symptoms, such as urinary frequency, dysuria, pelvic pain or discomfort, urinary retention, and hematuria (due to synchronous radiation cystitis [RC]) (22). After a meticulous anamnesis, the clinical diagnostic hypothesis should be confirmed by performing (procto)sigmoidoscopy. Endoscopic images usually reveal an edematous, hyperemic friable mucosa with telangiectasias and possibly areas of ulceration or sloughing (8, 14, 23, 24). Three major therapeutic approaches can be used in RIP – medical, endoscopic, and surgical – and there is no gold standard treatment regimen (8). Among pharmacological regimens, formalin application (4-10%) has been demonstrated to be an effective medical treatment (8, 23, 25–28), as well as sucralfate enemas (6, 23, 29), and metronidazole combined with ciprofloxacin (20, 29). Argon plasma coagulation (APC) is the first-line endoscopic strategy for individuals with hemorrhagic RIP (24, 29–31). Surgery is generally reserved for advanced stages of RIP refractory to optimal medical and endoscopic therapies (8, 30).

Hyperbaric oxygen therapy (HBOT) is another effective and safe intervention for managing RIP (24). It is characterized by inhaling pure oxygen (FiO_2_ 100%) inside a hyperbaric chamber frequently pressurized at 2.0 to 2.8 atmospheres absolute (ATA) for 60 to 120 minutes daily (8, 32, 33). This treatment leads to a series of physiological effects due to increased plasma oxygen concentration and delivery to tissues, contributing to better tissue oxygenation, enhancing microvascularization, and promoting angiogenesis. It also exerts a tumor sensitization effect on RT (34). It might be associated with mild and transient side effects due to pressure and oxygen toxicity, mostly middle ear barotrauma (MEB) (35, 36).

Currently, HBOT represents a primary and complementary standard treatment in several clinical scenarios, including late radiation tissue injuries (LRTIs), namely RIP and RC (type 1 recommendation, level B evidence) (36, 37). However, clinical evidence is still scarce. This retrospective study pretends to evaluate the effectiveness of the HBOT in treating RIP. Secondary objectives include assessing the hematochezia resolution rate; estimation of hematochezia recurrence; evaluation of endoscopic improvement; the correlation between response rates and the number of HBOT sessions; and finally, the evaluation of the HBOT safety level.

## 2 Materials and methods

### 2.1 Patients

**Figure 1:**
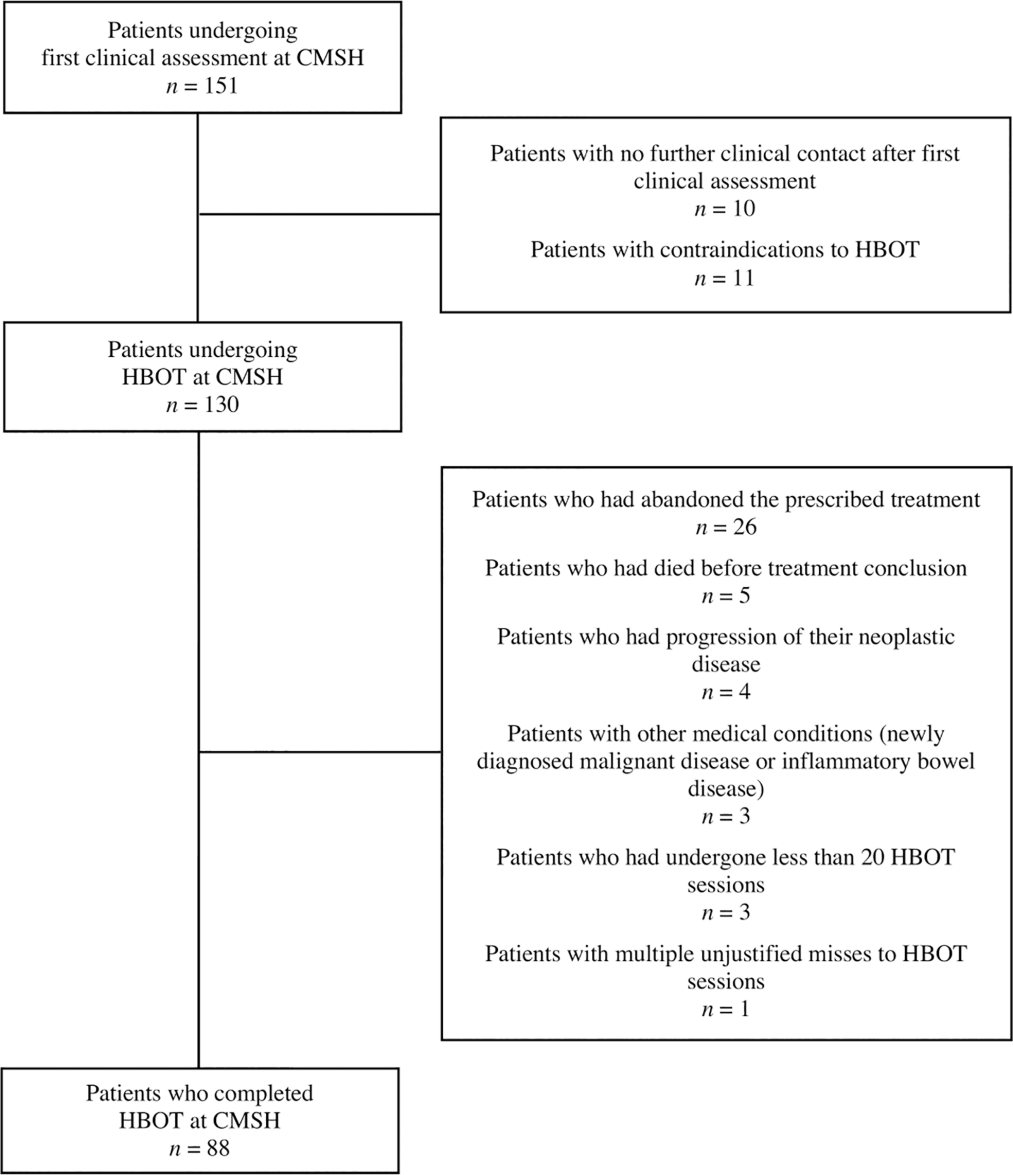
Flowchart exhibiting individuals eligible for this study and all excluded patients. After a preliminary screening, 88 patients were selected according to our inclusion and exclusion criteria and were further analyzed in this study. CMSH, Centro de Medicina Subaquática e Hiperbárica; HBOT, hyperbaric oxygen therapy.

This retrospective observational single-center study aims to evaluate the effectiveness of HBOT in a group of patients with late RIP treated within the past ten years (from January 2013 to March 2023) at Centro de Medicina Subaquática e Hiperbárica (CMSH), part of the Armed Forces Hospital (Lisbon, Portugal). **Figure 1** shows the flowchart of this study. After a preliminary screening, 88 patients were selected according to our inclusion and exclusion criteria and were further analyzed in this study.

This patient subgroup comprises individuals aged at least 18 years of age who have undergone RT as a curative treatment for pelvic cancer, considering multiple primary pelvic malignancies (e.g., colorectal, prostate, cervical, and uterine). The enrolled patients presented with clinical and/or radiological and/or histological evidence of RIP, including chronic diarrhea/constipation, abdominal cramp, proctalgia, lower gastrointestinal (GI) bleeding/hematochezia, obstruction, tenesmus, fecal urgency/incontinence, proliferative mucosal telangiectasias, ulceration, or development of fistulae. Patients with concurrent clinical manifestations resulting from other LRTIs (e.g., [entero]colitis, RC, and urethritis) were also included. Patients eligible for the study had a previous endoscopic examination with a diagnosis of rectal lesion. Also, eligible patients have failed at least one previous conservative treatment (pharmacological or endoscopic). Exclusion criteria included a number of HBOT sessions inferior to 20; patients who have refused/abandoned the post-HBOT follow-up period; patients with medical conditions that may justify a similar clinical presentation as LRTIs (e.g., inflammatory bowel disease, colorectal cancer); and patients with contraindications for HBOT (e.g., uncontrolled epilepsy, pneumothorax, severe claustrophobia, a recent episode of barotrauma).

To evaluate the effectiveness of HBOT, we establish the complete, partial, and overall response rates, as well as the non-response rate. The total resolution of symptoms defines a complete response; a partial response is characterized by a reduction of frequency and/or severity of symptoms; and non-response means the maintenance of symptoms as presented at the time of diagnosis or even its aggravation. Patients were considered improved in their RIP-related symptomatology if they have demonstrated a complete or partial response to HBOT. Our analysis also assessed the percentage of patients who had developed HBOT side effects and compliance with the proposed treatment protocol.

The Armed Forces Hospital ethics committee approved this study. Due to its retrospective nature and the fact that the clinical data could not identify the enrolled patients, informed consent was not required. Nevertheless, all clinical data were analyzed under confidentiality for the sole purpose of this study.

### 2.2 Treatment protocol

**Figure 2:**
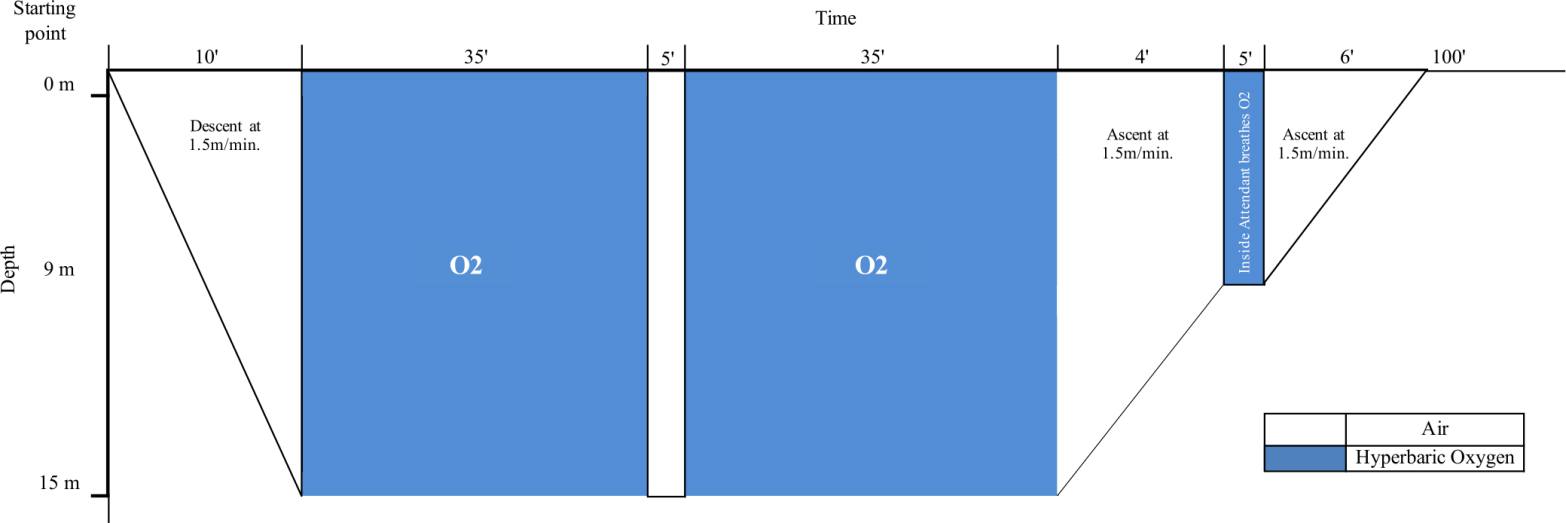
Treatment protocol for RIP at Centro de Medicina Subaquática e Hiperbárica (CMSH). Patients were treated inside a multiplace first class hyperbaric chamber, where they breathed 100% oxygen administered at 2.5 ATA, for 70-min periods (total duration of 100-min, considering descending, a 5-minute air-break, and ascending), once daily, 5 times a week. ATA, atmospheres absolute; RIP, radiation-induced proctitis.

The HBOT was performed at CMSH, part of the Armed Forces Hospital (Lisbon, Portugal). Patients were treated inside a multiplace first class hyperbaric chamber, where they were breathing 100% oxygen administered at 2.5 ATA, for 70-min periods (total duration of 100-min, considering descending, a 5-minute air-break, and ascending), once daily, 5 times a week, as shown in **Figure 2**. After the conclusion of an initial treatment course of 20 sessions, patients were evaluated to assess if there was an improvement in clinical signs and symptoms. The treatment ended if patients were revealed to be asymptomatic (mostly considering hematochezia). On the other hand, if patients remained symptomatic, the treatment protocol’s total duration might be extended to several weeks until a clinical and/or endoscopic best clinical response was obtained. Once treatment was concluded, patients were re-evaluated and referred to the attending physician, who had initially reported the case to our center and was responsible for the patient’s follow-up. In case of recurrence of symptoms, the patient had to be referred again to our center for further evaluation.

Patients were submitted to a prior clinical evaluation to assess their eligibility to initiate the treatment protocol, ruling out significant contraindications to HBOT. Before the initiation of HBOT, it was also mandatory to perform: a chest radiologic examination (to exclude pneumothorax, pulmonary blebs or bullae, or other relevant pulmonary diseases), a standard 12-lead electrocardiogram (to exclude major cardiac pathology), and a tympanogram (to exclude Eustachian tube dysfunction). Those individuals also received indications from the nursing team about the precautions to take prior to and after each treatment session to avoid complications.

### 2.3 Data collection

All clinical data were retrospectively collected and analyzed by revisiting the patients’ medical records archived at CMSH installations. The accessed clinical data include the type, time of diagnosis, and corresponding treatment of the pelvic organ cancer; clinical manifestations, time of diagnosis, and previous treatments of RIP and other synchronous LRTIs; and all the relevant data related to the response and compliance to HBOT.

### 2.4 Statistical analysis

The data analysis was performed in Microsoft Excel and Statistical Package for the Social Sciences (IBM^®^ SPSS^®^ Statistics 23.0) software.

In addition to descriptive statistics, Chi-square or Fisher exact tests were used to compare categorical data; the Mann-Whitney U or Kruskal-Wallis tests to compare continuous/ordinal data between two or more groups of a nominal independent variable, respectively; the Spearman’s correlation analysis to relate continuous/ordinal data to each other; and logistic regression to predict binary or multinomial outcomes given a set of independent continuous/ordinal variables. Normal distribution for all continuous variables was tested using the Kolmogorov-Smirnov test. A p-value <0.05 was considered to indicate statistical significance (two-tailed).

## 3 Results

### 3.1 Sample characterization

A total of 151 patients with RIP were referred to CMSH to be treated with HBOT. Twenty-one of them did not start the treatment because there was no further clinical contact after the first clinical assessment (n=10) or because the HBOT was contraindicated due to active neoplastic disease (n=6), major cardiac pathology (n=2), a recent episode of barotrauma/pathologic tympanogram (n=1), pulmonary blebs (n=1), or relevant cognitive impairment conditioning the compliance to HBOT (n=1). Additionally, 42 patients were excluded after HBOT initiation. This subgroup comprised patients who had abandoned the prescribed treatment/failed additional HBOT prescribed sessions (n=26); patients who had died before treatment conclusion (n=5); patients who had documented progression of their neoplastic disease (n=4); patients with other medical conditions that were the actual cause of GI bleeding (e.g., newly diagnosed malignant disease [n=2] and inflammatory bowel disease [n=1]); patients who had undergone less than 20 HBOT sessions (n=3); and a patient who had multiple unjustified misses to HBOT sessions (n=1).

Thus, after excluding 63 patients, this retrospective study analyzed 88 patients undergoing complete treatment with HBOT for RIP from January 2013 to March 2023, as shown in **Figure 1**.

Considering the population evaluated in this study, 83% of them were male patients (n=73) and 17% female patients (n=15), with a median age of 69.5 (interquartile range [IQR]: 64-75). The primary tumor most frequently reported justifying the need for RT was prostate cancer in 77.3% (n=68), and the second most frequent malignancy was *cervix uteri* cancer, with 10.2% incidence (n=9). Also, 78.4% of patients had undergone external beam radiation therapy (n=69), 10.2% brachytherapy (n=9), and 11.4% both RT approaches (n=10). Nevertheless, 52.3% had at least performed another oncological treatment, namely surgery, androgen deprivation therapy, and chemotherapy. Furthermore, 61.4% of patients had received the diagnosis of RIP with not having other synchronous LRTIs (n=54). The second most common diagnosis combined RIP and RC, representing 30.7% of the analyzed population (n=27). **Table 1** describes demographic and clinical data related to the primary tumor, type of RT, patient’s clinical presentation, and previous therapeutic approaches for RIP.

**Table 1:** Sample characterization, considering patient’s sex, type of primary malignant tumor, implemented oncological treatments, LRTI diagnosis (other than RIP), patient’s clinical presentation at our first clinical assessment, and previous therapeutic approaches for RIP.

Variable	% (n)
Sex	
Female	17 (15)
Male	83 (73)
Primary malignant tumor	
Anal canal	3.4 (3)
* Cervix uteri*	10.2 (9)
Colon	1.1 (1)
* Corpus uteri*	3.4 (3)
Prostate	77.3 (68)
Rectum	3.4 (3)
Rectum + prostate	1.1 (1)
RT treatments	
BT	10.2 (9)
EBRT	78.4 (69)
EBRT + BT	11.4 (10)
Other oncological treatments	
No	47.7 (42)
Yes	52.3 (46)
Type of other oncological treatments	
ADT	18.2 (16)
ADT + surgery	2.3 (2)
CTX	8.0 (7)
CTX + ADT + surgery	1.1 (1)
CTX + surgery	4.5 (4)
Surgery	18.2 (16)
LRTI diagnosis	
RIP isolated diagnosis	61.4 (54)
RIP plus other LRTI diagnosis	38.6 (34)
RIP + colitis + enteritis	2.3 (2)
RIP + cystitis	30.7 (27)
RIP + cystitis + enteritis	1.1 (1)
RIP + cystitis + enteritis + urethritis	1.1. (1)
RIP + cystitis + urethritis	1.1 (1)
RIP + enteritis	2.3 (2)
Hematochezia	
No	13.6 (12)
Yes	86.4 (76)
Intensity of blood loss	
Mild/sporadic (CTCAE Grade 1)	28.1 (16)
Moderate/intermittent (CTCAE Grade 2)	8.8 (5)
Intense/daily/requiring a red blood cell concentrate transfusion (CTCAE Grade ≥3)	63.1 (36)
Need for transfusion therapy	
No	72.7 (64)
Yes	27.3 (24)
Other clinical manifestations	
No	38.6 (34)
Yes	61.4 (54)
Symptoms/signs	
Abdominal cramp	5.7 (5)
Constipation	4.5 (4)
Diarrhea	10.2 (9)
Fecal incontinence/urgency	9.1 (8)
Hematuria	28.4 (25)
LUTS (dysuria/pollakiuria/retention/urgency/incontinence)	11.4 (10)
Meteorism	2.3 (2)
Mucorrhea	1.1 (1)
Proctalgia	15.9 (14)
Tenesmus	18.2 (16)
Ulceration/fistulae/fissure	11.4 (10)
Pre-HBOT endoscopic treatment (APC)	
No	52.3 (46)
Yes	47.7 (42)

ADT, androgen deprivation therapy; APC, argon plasma coagulation; BT, brachytherapy; CTCAE, Common Terminology Criteria for Adverse Events; CTX, chemotherapy; EBRT, external beam radiation therapy; HBOT, hyperbaric oxygen therapy; LRTI, late radiation tissue injury; LUTS, lower urinary tract symptoms; RIP, radiation-induced proctitis; RT, radiotherapy.

### 3.2 Clinical presentation

The median time between the end of RT and RIP-related symptomatology initiation was 12 months (IQR: 7.25-24). Hematochezia represents the most common clinical manifestation of RIP in this group of patients, emerging in 86.4% of the patients (n=76). Among the 13.6% of individuals whose most preeminent clinical manifestation was not hematochezia (n=12), only 25% received the isolated diagnosis of RIP (n=3), presenting other nonspecific GI symptoms. Regarding the intensity of GI blood loss, 28.1% of patients developed mild/sporadic hematochezia (CTCAE Grade 1), 8.8% experienced moderate/intermittent hematochezia (CTCAE Grade 2), and 63.1% developed intense/daily hematochezia or severe anemia requiring transfusion support (CTCAE Grade ≥3). Considering the total number of patients, around 30% had required at least one red blood cell concentrate transfusion (n=24). About other clinical manifestations, the most frequently reported were hematuria in 28.4% of cases, tenesmus (18.2%), proctalgia (15.9%), and the development of ulceration/fistulae/fissure (11.4%).

It is also important to reinforce that most patients (91%) had documented a previous endoscopic examination confirming the RIP diagnosis (only 8 clinical records did not have registered any information concerning pre-HBOT endoscopic evaluation). All patients tried at least one last conservative treatment modality (pharmacological or endoscopic treatments) that was revealed to be ineffective. Almost half of the patients (47.7%) underwent at least one APC treatment session before starting HBOT. Although only 35 clinical registries confirmed the attempt to treat RIP with medical treatment at the beginning, some pharmacological treatment has been carried out as it is classically the first line treatment to be applied in the management of RIP.

### 3.3 Response to hyperbaric oxygen therapy

**Figure 3:**
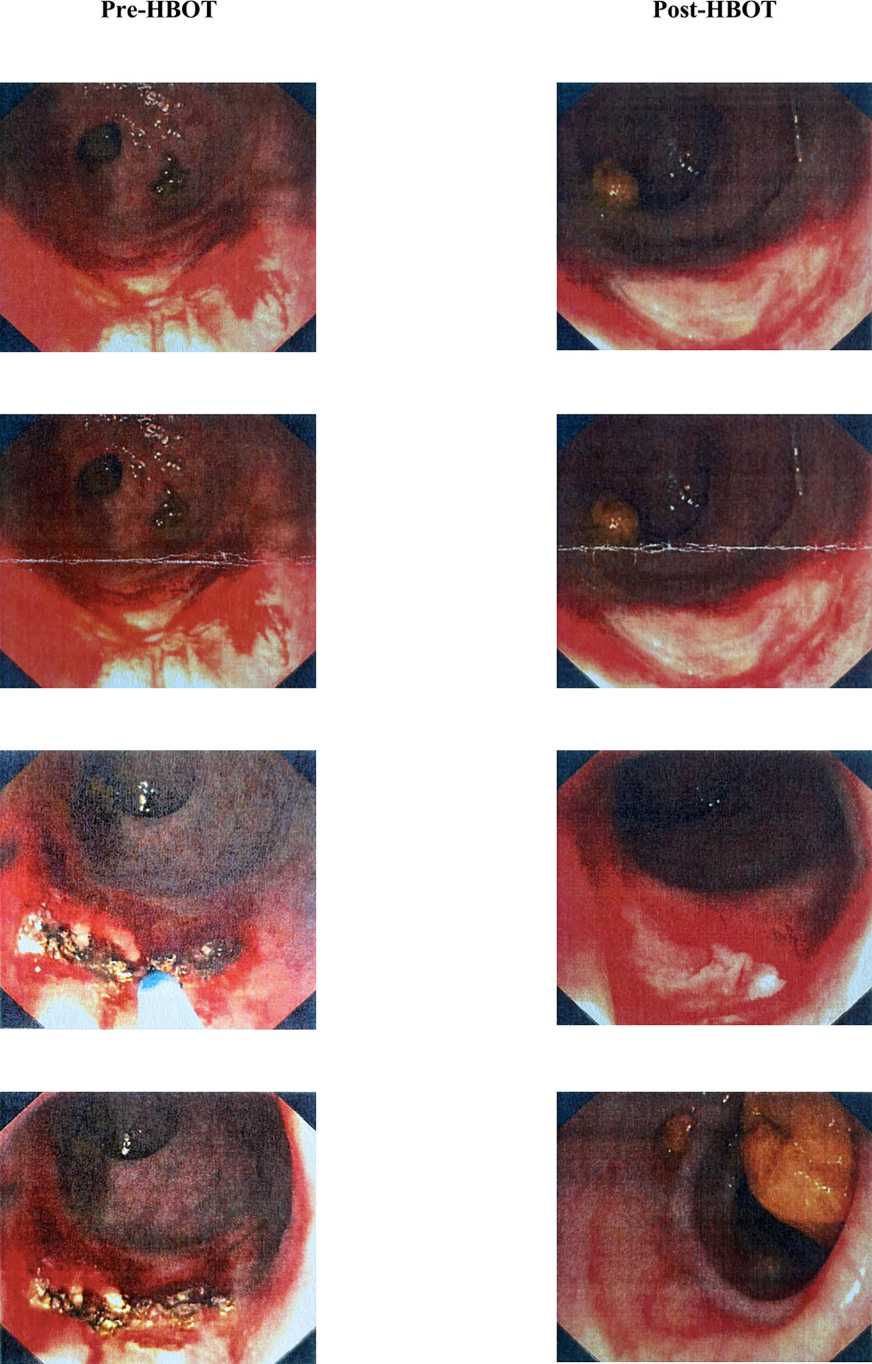
Pre- and post-HBOT endoscopic findings of one patient. Left side (pre-HBOT): just-anal rectal mucosae bleeding actively, compatible with RIP secondary to BT; APC was performed to control bleeding telangiectasias. Right side (post-HBOT): visualization of 3cm-superficial ulceration on the just-anal rectal mucosae, with friability and cicatricial areas, after 60 HBOT sessions. APC, argon plasma coagulation; BT, brachytherapy; HBOT, hyperbaric oxygen therapy; RIP, radiation-induced proctitis.

The median time between installing RIP-related symptomatology and initiating HBOT was 8 months (IQR: 3.25-14). Patients completed a median of 60 HBOT sessions (IQR: 40-87.5) during a median time of 5.82 months (IQR: 3.07-11.24). An overall response rate of 94.3% was revealed – 62.5% of patients had a complete clinical response with full resolution of symptoms, and 31.8% had a partial clinical response with a reduction of frequency or severity of symptoms (e.g., patients reported the occurrence of less frequency and quantity of GI blood loss to isolated episodes of hematochezia, with no hemodynamic repercussions). The registered hematochezia resolution rate was 93.7% (considering both complete and partial resolution). Individuals with at least two concomitant LRTIs (e.g., RIP and RC) were associated with a complete response to HBOT (p=0.029). Response to HBOT showed no differences according to gender, age, primary tumor, type of RT, whether other cancer treatments were performed or not, the initial symptoms, the time between the end of RT and the onset of symptoms, the time between symptoms and HBOT, and the need for blood transfusions or treatments prior to HBOT. Only about 5.7% of patients did not respond to the treatment, maintaining refractory hematochezia (6.3%) or the other initial symptoms. Also, 34.1% of patients performed a post-HBOT endoscopic evaluation, and 83.3% showed endoscopic improvement of RIP, as shown in **Figure 3**. The time between the end of RT and the onset of hematochezia was inversely associated with the number of HBOT sessions. Although, the relationship is weak because Spearman’s rho (ρ) correlation coefficient is between -0.20 and -0.29 (ρ: -0.259; p=0.036).

Sample characterization considering refractory symptoms, post-HBOT endoscopic findings, clinical response to HBOT and its related side effects, hematochezia relapse, and the need to perform a surgical procedure for symptomatic control are listed in **Table 2**. It is also important to note that in about 21.6% (n=19) of cases there were minor complications related to HBOT, and the most frequently reported adverse event was treatable MEB (20.5%; n=18). Only one case of reversible myopia was reported, and no other side effects were observed during or after the treatment protocol conclusion. The number of HBOT sessions was a statistically significant predictor of HBOT side effects (odds ratio= 1.010; 95% confidence interval, 1.000-1.020; p=0.047).

**Table 2:** Sample characterization considering clinical response rates, refractory symptoms, post-HBOT endoscopic exam results, side effects development, hematochezia recurrence, and the necessity to perform a surgical procedure for symptomatic control.

Variable	% (n)
Clinical response	
Overall response rate	94.3 (83)
Complete response rate	62.5 (55)
Partial response rate	31.8 (28)
No response rate	5.7 (5)
Resolution of hematochezia	93.7 (74)
Complete resolution	64.6 (51)
Partial resolution	29.1 (23)
Refractory hematochezia (no resolution)	6.3 (5)
Other refractory symptoms	15.9 (14)
Recurrence of hematochezia	14.8 (13)
Post-HBOT endoscopic exam	
Yes	34.1 (30)
No	65.9 (58)
Post-HBOT endoscopic findings	
Endoscopic improvement	83.3 (25)
Endoscopic maintenance	13.3 (4)
Endoscopic aggravation	3.3 (1)
Side effects	
Yes	21.6 (19)
No	78.4 (69)
Type of side effect	
Middle ear barotrauma	20.5 (18)
Reversible myopia	1.1 (1)
Necessity to perform a surgical procedure	
Yes	5.7 (5)
No	94.3 (83)

HBOT, hyperbaric oxygen therapy.

The estimated hematochezia recurrence rate was 14.8% (n=13), considering the progression of the disease. In 5.7% of cases, a surgical procedure to achieve symptomatic control or fistulae formation resolution was needed (n=5). The average follow-up time at our center was 1.8 months. Considering hematochezia as the most prevalent clinical manifestation (86.4%), we further analyzed in detail the exclusive relationship between hematochezia resolution rate and refractory hematochezia rate with the other most relevant variables, both categorical (sex, primary tumor, type of radiation-induced soft tissue lesions, need for transfusion support, pre-HBOT APC treatment, post-HBOT endoscopic results) and continuous (time between RT and symptoms onset, the time between symptoms onset and HBOT initiation, number of HBOT sessions and total duration of HBOT). No statistically significant relations were found, as specified in **Table 3**; however, there is a trend to achieve hematochezia resolution (complete or partial) after completing at least 70 HBOT sessions (p=0.075, applying Fisher’s exact test). While partial and complete responses require fewer than 70 sessions of HBOT in terms of overall RIP symptoms (p=0.069), isolated hematochezia tends to require at least 70 sessions by Fisher’s exact test.

**Table 3:** Relationship between hematochezia resolution rates *versus* other categorical and continuous variables (after categorization).

Variable	Hematochezia, %(n)		
	Resolution	No resolution	P-value
Sex			
Male (n=66)	93.9 (62)	6.1 (4)	>0.05
Female (n=13)	92.3 (12)	7.7 (1)	
Primary tumor			
Prostate (n=62)	93.5 (58)	6.5 (4)	0.465
*Cervix uteri* (n=8)	87.5 (7)	12.5 (1)	
*Corpus uteri* (n=3)	100	0	
Rectum (n=2)	100	0	
Anal canal (n=2)	100	0	
Colon (n=1)	100	0	
Rectum + prostate (n=1)	100	0	
LRTI diagnosis			
RIP (n=52)	92.3 (48)	7.7 (4)	>0.05
RIP + RC (n=21)	95.2 (20)	4.8 (1)	
RIP + enteritis (n=2)	100	0	
RIP + enteritis + colitis (n=1)	100	0	
RIP + RC + enteritis (n=1)	100	0	
RIP + RC + urethritis (n=1)	100	0	
RIP + RC + enteritis + urethritis (n=1)	100	0	
Inflammation in more than one organ			
No (n=52)	92.3 (48)	7.7 (4)	0.656
Yes (n=27)	96.3 (26)	3.7 (1)	
Need for transfusion therapy			
No (n=57)	96.5 (55)	3.5 (2)	0.129
Yes (n=22)	86.4 (19)	13.6 (3)	
Pre-HBOT APC treatment			
No (n=37)	97.3 (36)	2.7 (1)	0.364
Yes (n=42)	90.5 (38)	9.5 (4)	
Time between RT and hematochezia			
≤12 months (n=35)	94.3 (33)	5.7 (2)	>0.05
13-24 months (n=15)	93.3 (14)	6.7 (1)	
>24 months (n=16)	93.8 (15)	6.2 (1)	
Time between hematochezia and HBOT			
≤3 months (n=18)	100 (18)	0 (0)	0.432
4-12 months (n=34)	88.2 (30)	11.8 (4)	
>12 months (n=21)	95.2 (20)	4.8 (1)	
Time of HBOT (months)			
≤2 month (n=13)	100 (13)	0 (0)	0.574
3-6 months (n=29)	89.7 (26)	10.3 (3)	
>6 months (n=37)	94.6 (35)	5.4 (2)	
Number of sessions			
≤20 sessions (n=6)	100 (6)	0 (0)	0.236
21-70 sessions (n=42)	97.6 (41)	2.4 (1)	
>70 sessions (n=31)	87.1 (27)	12.9 (4)	

P-values were obtained with Chi-square tests (Fisher’s exact test). Abbreviations: APC, argon plasma coagulation; HBOT, hyperbaric oxygen therapy; LRTI, late radiation tissue injury; RC, radiation cystitis; RIP, radiation-induced proctitis; RT, radiotherapy.

## 4 Discussion

Consistent guidelines are not currently available for the management of RIP (24). Generally, patients are treated empirically on a tailored basis according to patient comorbidities, the severity of the clinical condition, and medical and institutional expertise (6, 8, 16, 24).

The HBOT is used in several clinical conditions as well as in professional and military training (8, 37, 38). As a result of the 10^th^ European Consensus Conference on Hyperbaric Medicine, HBOT was recommended for the treatment of soft tissue radionecrosis, namely, RIP and RC (type 1 recommendation, level B evidence) (36). The first cutting-edge case report in this setting was conducted by Charneau J et al. (1991). It revealed the utility of HBOT in treating a 74-year-old male patient with a history of transfusion-dependent hemorrhagic RIP refractory to medical treatment. This patient achieved a complete response after 82 HBOT sessions (2.5 ATA, for 90 min, twice daily), with the clinical response remaining for at least 9 months, avoiding colostomy (39). Afterward, it was the motto for several case reports and small series, retrospective studies, clinical trials, reviews, and meta-analyses published in the last three decades (40–68).

In a Cochrane systematic review, Bennett M et al. (2016) reviewed 14 studies that showed moderate-quality evidence of HBOT in the management of LRTIs, including RIP (40). Another earlier systematic review conducted by Feldmeier J and Hampson NB (2002) considered 74 publications (including 14 on RIP and radiation enteritis), in which all but seven reported a positive outcome. Thus, based on the consistency of these findings, HBOT was considered “Likely to be Beneficial” for the treatment of patients with RIP and radiation enteritis (41).

We report real-world evidence of HBOT effectiveness in a large cohort of patients (n= 88) with RIP over a significant period of time (10 years).

Achieving an overall clinical response and hematochezia resolution rates of 94.3% and 93.7%, respectively, we can state that HBOT proved effective in managing RIP. These numbers reinforce the importance of this study compared to others, although positive, have less statistical robustness (40–46). The HOT2 clinical trial was an exception, which controversially demonstrated that HBOT was ineffective in treating chronic bowel dysfunction following pelvic RT (44). However, several limitations were pointed out for this study, such as the small sample size for a phase 3 trial, the predefined follow-up duration of only 12 months or even the exclusion of patients with grade 3 symptoms of LENT-SOMA score (including severe fecal incontinence and transfusion-dependent RIP) (8).

Generally, there is an underestimation of RIP mild symptoms, either by patients or physicians (8, 46). As expected, hematochezia represented our series’ most common clinical manifestation of RIP. The reason for this is possibly related to the fact that hematochezia is the most appreciated symptom and the primary manifestation of RIP, as it is an alarming symptom for the doctor-patient binomial. This information is consistent with data from previous studies, including that by Clarke RH et al. (2008), with the majority of patients presenting lower GI hemorrhage and other chronic symptoms such as diarrhea, constipation, ulceration, and pain (47). Additionally, our study showed a higher hematochezia resolution rate (complete and partial) when compared to other studies, such as that by Dall’Era MA et al. (2006) and Oliai C et al. (2012) with 76% and 75%, respectively (49, 50).

Furthermore, some patients have experienced symptoms other than GI as a result of RT damage to surrounding healthy tissues (22). Tolerance to irradiation of pelvic structures varies from specific organ to another. Normal tissues of the vagina, cervix, and uterus can withstand high radiation doses and recover quickly from RT lesions (51, 52). On the opposite side, the bladder, sigmoid, rectosigmoid, and rectum are more prone to radiation toxicity. Utmost point doses of 75 and 70 Gy are acceptable for the bladder and rectum, respectively (51, 53, 54). This supports that RIP and RC are two commonly reported LRTIs, possibly arising concurrently (51, 54–56). In fact, the combination of RIP and RC was our second most common LRTI diagnosis. A previous study conducted by Ribeiro de Oliveira T et al. (2015) evaluating hemorrhagic RC response to HBOT pointed to 23.9% of patients with LRTIs other than RC (namely RIP and enteritis) (55). Our data showed that individuals with at least two concurrent LRTIs were associated with complete response to HBOT, probably because other LRTIs require fewer HBOT sessions to achieve a complete clinical resolution and, consequently, are easier to treat when compared to RIP. Furthermore, previous studies investigating the efficacy of HBOT in treating RC, such as that by Oscarsson N et al. (2019), demonstrated that 30 HBOT sessions were associated with clinically relevant improvement in RC (57). Ribeiro de Oliveira T et al. (2015) also showed that 89.6% of patients achieved complete resolution of hematuria after an average of 37 sessions (55). Similarly, Degener S et al. (2012) presented an 80% complete resolution of hematuria after 30 sessions for a 35-month follow-up (58). Therefore, we believe that this treatment modality should be specially considered as a first-line treatment for patients who develop more than one LRTI (55, 59).

Patients eligible for our study tried at least one last conservative treatment modality (medical or endoscopic) that proved to be ineffective in treating RIP-associated symptomatology. Besides only 35 clinical records confirm the attempt to treat RIP initially with medical treatment, we believe that some pharmacological treatment has been performed globally as it is classically the first-line treatment to be applied in the management of RIP (8, 23, 29, 69). Sucralfate and mesalamine enemas were the most frequently prescribed treatments; less common options included metronidazole, vitamin A, and corticosteroid enemas. About 50% of patients underwent APC treatment before starting HBOT. Álvaro-Villegas JC et al. (2011) found that APC and HBOT were equally effective in treating RIP with regard to controlling rectal bleeding, the need for transfusions, and the impact on tissue toxicity. However, APC was associated with faster and higher responses at baseline (60). One of the relevant aspects of our series is that we have a heavily treated population in which HBOT played an important role in its subsequent control.

Additionally, our data showed that the time between RT and the onset of hematochezia was inversely associated with the number of HBOT sessions required. Therefore, it can be inferred that patients with a longer time until the clinical manifestation of hematochezia may have a better prognosis. After completing a median of 60 sessions, the overall response to HBOT tended to be inversely associated with the number of sessions, possibly because patients with a better response to HBOT needed fewer sessions. However, there was a trend for hematochezia resolution after completing at least 70 HBOT sessions. In general, the number of sessions performed in our study was higher than in other studies that present a full course treatment with an average of 40 sessions (42, 49, 61). This higher number of prescribed sessions may be explained by the fact that we treat more severe cases of RIP, as patients come to us in later phases and, consequently, with more refractory symptoms. Hence, due to our institutional experience, we anticipate the need for a more prolonged treatment, frequently surpassing 40 sessions.

Although rare and usually non-severe, HBOT has known potential adverse events. In a retrospective analysis of 1.5 million treatments, only 0.68% were associated with a side effect, being barotrauma and confinement anxiety the most reported events (62). The incidence of MEB varies according to the series and ranges from 0.37 to 84% in non-ventilated patients versus 94% in ventilated patients (38, 63, 64). Our data align with published data and showed that the number of HBOT sessions was a predictor of side effects, mainly MEB.

Lastly, our retrospective study is not exempted from limitations. The current analysis is limited by several factors, including its retrospective nature, the single institution source of data, and the quality of our medical and supervisor records compromised by the lack of complete clinical information. Once patients turned asymptomatic (mainly in relation to hematochezia) and completed the treatment course, they were referred to the attending physician. One of the limitations is the short follow-up period and the loss of follow-up of treated patients after referral to the attending physician. These facts may have prevented more reliable monitoring of symptoms or even their relapse. Moreover, it may even influence the evaluation of the hematochezia recurrence rate. The lack of grading of adverse events based on a validated classification did not allow for drawing more robust conclusions, configuring another important limitation of our study. The missed information about the different RT protocols administered (total dose and fractionation) and comprehensive information related to pre-HBOT medical treatment conditioned our analysis of the potential relationship between different variables and the clinical response to HBOT. Also, the inexistence of a more detailed characterization of the post-HBOT clinical picture limited us from assessing the remaining symptoms.

In this cohort of HBOT-treated refractory RIP patients (the largest reported to date), the overall clinical response rate was 94.3% (including 62.5% complete responses) after a median of 60 HBOT sessions. Furthermore, HBOT proved to be a safe treatment when patients are previously studied and the correct protocol is applied. This real-world evidence study adds value to published data on the management of RIP with HBOT.

## Data availability statement

The original contributions presented in the study are included in the article/supplementary material. Further inquiries can be directed to the corresponding authors.

## Ethics statement

The studies involving human participants were reviewed and approved by Armed Forces Hospital Ethics Committee - Armed Forces Hospital, Lisbon, Portugal. Written informed consent for participation was not required for this study in accordance with the national legislation and the institutional requirements.

## Author contributions

The present manuscript is the result of the original work by the authors. AMM and DAC conceived and designed the study. AMM performed the consultation of medical records. AMM, DAC, and VM performed the analysis and interpretation of the data; all the authors approved manuscript draft. DAC, VM, and CEA performed the manuscript supervision.
